# A novel WT1 gene mutation in a chinese girl with denys‐drash syndrome

**DOI:** 10.1002/jcla.23769

**Published:** 2021-05-04

**Authors:** Faliang Wang, Jiabin Cai, Jinhu Wang, Min He, Junqing Mao, Kun Zhu, Manli Zhao, Zhonghai Guan, Linjie Li, Hongchuan Jin, Qiang Shu

**Affiliations:** ^1^ Department of Surgical Oncology Children’s Hospital National Clinical Research Center for Child Health Zhejiang University School of Medicine Hangzhou China; ^2^ Department of Pathology Children’s Hospital National Clinical Research Center for Child Health Zhejiang University School of Medicine Hangzhou China; ^3^ Laboratory of Cancer Biology, Key Lab of Biotherapy in Zhejiang Sir Run Run Shaw Hospital Zhejiang University School of Medicine Hangzhou China

**Keywords:** 46, Denys‐Drash syndrome (DDS), nephrotic syndrome, Wilms tumor, Wilms tumor‐1 (*WT1*), XY karyotype

## Abstract

**Objective:**

Denys‐Drash syndrome (DDS) is defined by the triad of Wilms tumor, nephrotic syndrome, and/or ambiguous genitalia. Genetic testing may help identify new gene mutation sites and play an important role in clinical decision‐making.

**Methods:**

We present a patient with an XY karyotype and female appearance, nephropathy, and Wilms tumor in the right kidney. Genomic DNA was extracted from peripheral blood cells according to standard protocols. “Next‐generation” sequencing (NGS) was performed to identify novel variants. The variant was analyzed with Mutation Taster, and its function was explored by a cell growth inhibition assay.

**Results:**

We found the first case of Denys‐Drash syndrome with the uncommon missense mutation (c.1420C>T, p.His474 Tyr) in the *WT1* gene. In silico analysis, the variant was predicted “disease‐causing” by Mutation Taster. The mutated variant showed a weaker effect in inhibiting tumor cells than wild‐type *WT1*.

**Conclusions:**

The uncommon missense mutation (c.1420C>T, p.His474 Tyr) in the *WT1* gene may be a crucial marker in DDS.

## 1 INTRODUCTION

DDS is a rare genetic disorder of sex development featuring a triad of Wilms’ tumor, nephrotic syndrome, and the 46, XY karyotype. DDS is correlated with heterozygous missense mutations in the zinc‐finger (ZF) region of the *WT1* gene, which is a tumor suppressor gene involved in gonadal development and is located in chromosome band 11p13.^1^, ^2^

The *WT1* gene encodes a zinc‐finger transcription factor that is important for the normal development of the genital tract and the kidneys.^3^ Genitourinary defects were found in *WT1* knockout mice that died on embryonic day 13.5.^4^ The *WT1* gene comprises ten coding exons. The first six exons encode a glutamine‐ and proline‐rich N‐terminal region containing a potential RNA recognition motif, involved in self‐association, localization to nuclear speckles, transcriptional activation and repression, and nuclear export. The last four exons are also crucial for the function of *WT1*. They encode a ZF domain that plays a key role in DNA/RNA binding and nuclear localization.^5^, ^6^ Mutations in exons 8 or 9 are very common in DDS patients.^7^

**FIGURE 1:**
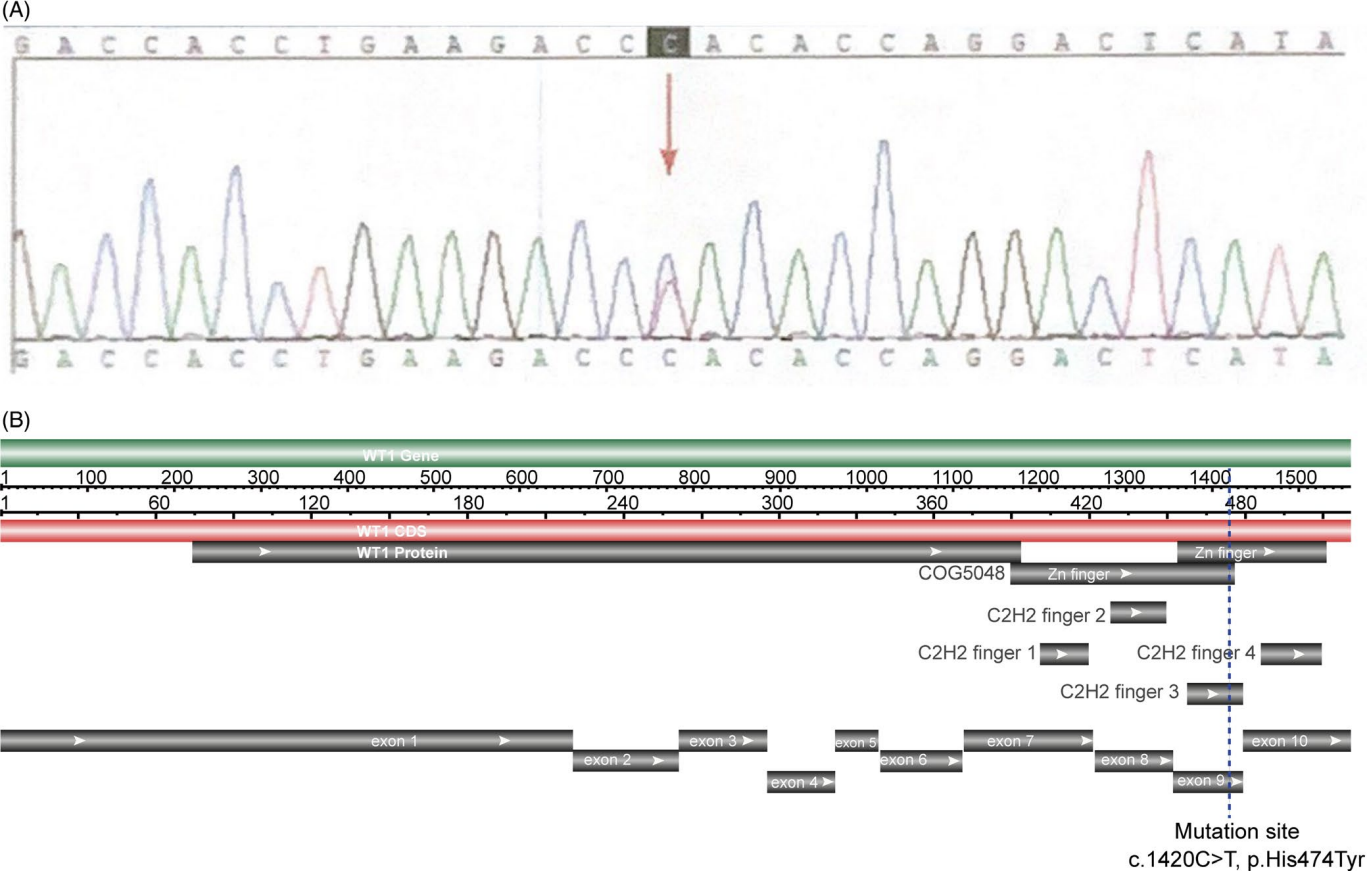
A, Sanger sequencing chromatogram shows the double peak C/T in WT1 gene. B, The location of the mutation site detected in the Coding DNA Sequence (CDS) of *WT1*. The NCBI Reference number is NM_024424.5

In this study, we present a patient with Wilms tumor, a female appearance, and a 46, XY karyotype who had previously unreported mutations in exon 9 in the ZF3 region of *WT1* (Figure 1).

## 2 METHODS

### 2.1 Participants

**FIGURE 2:**
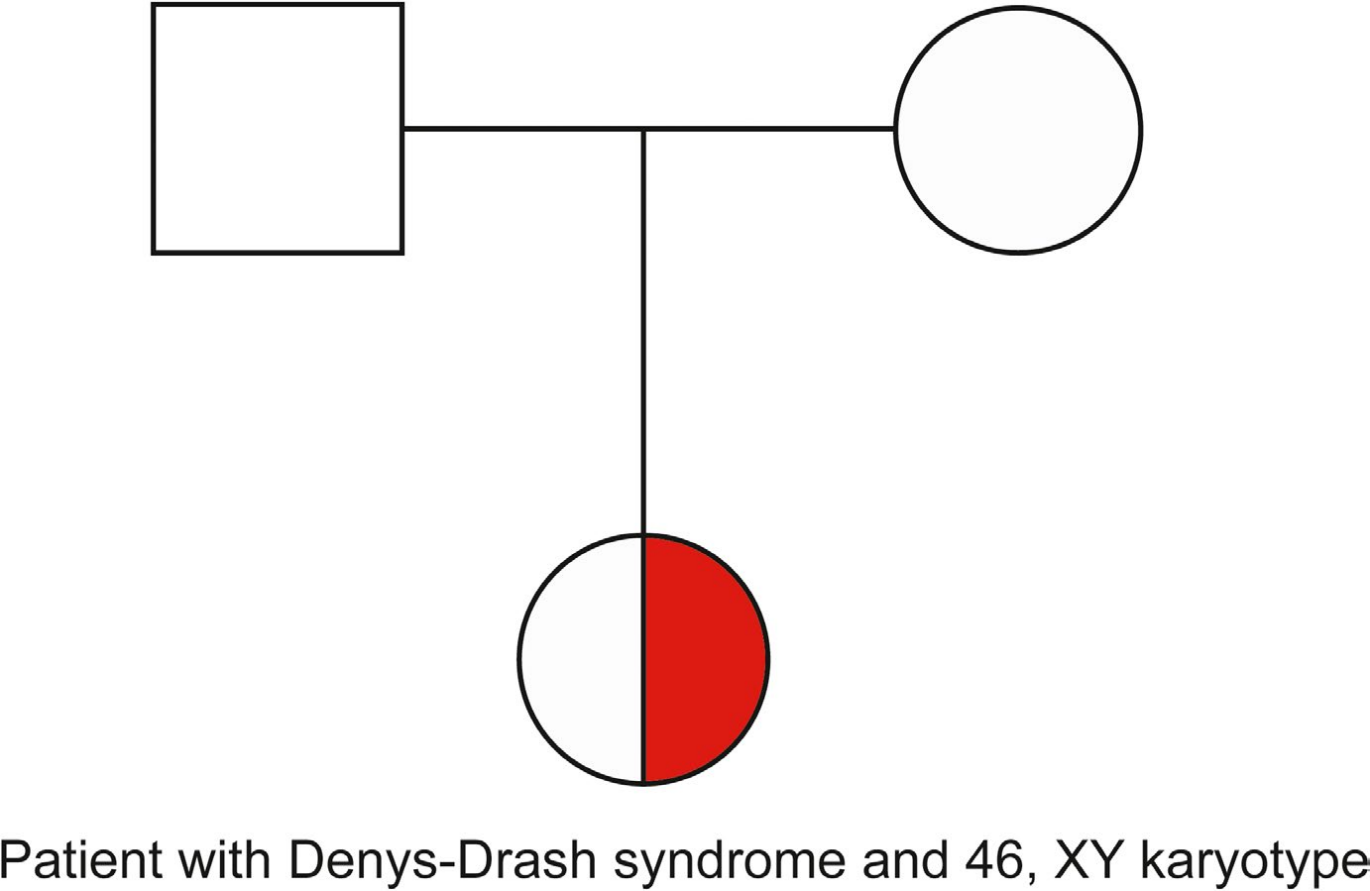
A pedigree diagram illustrating the heterozygous missense mutation in *WT1* gene in the patient's family. The half‐filled symbol indicates the proband (heterozygous carrier)

A 23‐month‐old girl with DDS and her parents from Zhejiang Province were enrolled in this study at the Department of Surgical Oncology, Children's Hospital, Zhejiang University School of Medicine, Hangzhou, Zhejiang, China (Figure 2). The study was approved by the ethics committee of Children's Hospital, Zhejiang University School of Medicine, Hangzhou, China, and the participants and guardians provided written informed consent in accordance with the recommendations of the Declaration of Helsinki.

### 2.2 Clinical assessment

A comprehensive clinical examination of the abdominal mass was performed. We conducted an ordinary physical examination, laboratory tests [routine blood, urine and fecal, liver and kidney function, electrolyte, tumor makers, and sex‐determining region on the Y chromosome (SRY)], and imageological examinations [electrocardiogram, abdominal B‐ultrasonography examination, and contrast‐enhanced computed tomography (CT)]. After that, a puncture biopsy was performed.

### 2.3 Clinical treatment

Based on the diagnosis of nephroblastoma (mesenchymal type), the patient was treated with chemotherapy, arterial interventional therapy, and nephroblastoma radical resection. Detailed information is summarized in Table 1.

**TABLE 1:** Summary of the treatment course of the patient

Time	Treatment
2015.09.29	Embolization and chemotherapy via the right femoral artery (Cisplatin +Pirarubicin + Vinorelbine)
2019.11.05	Vinorelbine +Ifosfamide + Etoposide
2015.11.20	Laparotomy, right nephroblastoma resection, lymphadenectomy, and partial adrenalectomy
2015.11.30	Vinorelbine +Pirarubicin + Carboplatin
2015.12.29	Vinorelbine +Ifosfamide + Etoposide
2016.01.22	Vinorelbine +Pirarubicin + Carboplatin
2016.03.03	Vinorelbine +Actinomycin D
2016.03.29	Vinorelbine +Carboplatin + Pirarubin
2016.4.28	Vinorelbine +Actinomycin
2016.5.25	Vinorelbine +Pirarubicin
2016.6.23	Vinorelbine +Actinomycin D
2016.07.23	Vinorelbine +Pirarubicin

### 2.4 Genetic testing

The identification process is a two‐step procedure: High‐throughput sequencing technologies and validation of suspected disease‐causing mutations (Sanger sequencing).

#### 2.4.1 High‐throughput sequencing

To make a precise diagnosis, we performed targeted “Next‐generation” sequencing (NGS) of 175 disease‐related genes. Written informed consent of the whole family was obtained prior to the collection of 5 mL of their peripheral blood for the following experiment. The study was approved by the ethics committee of the Beijing Genomics Institute (BGI).

A capture panel (NimbleGen, Madison, USA) of disease‐related genes was previously designed and assessed by our group. The capture panel comprised 2,433,298 bp that covered all exons together with the flanking exon and intron boundaries (±15 bp) of 175 disease‐related genes.

Genomic DNA was extracted from peripheral blood mononuclear cells using the QIAamp DNA Blood Midi Kit (Qiagen, Hilden, Germany) according to the manufacturer's instructions. Genomic DNA was fragmented by Covaris LE220 (Massachusetts, USA). DNA fragments were ligated to platform‐specific adaptors to generate DNA library (200–250 bp). The library was enriched by array hybridization according to previously published procedure,^8^ followed by elution and post‐capture amplification. The products were then subjected to Agilent 2100 Bioanalyzer and ABI StepOne to estimate the magnitude of enrichment. After quality control, captured library sequencing was carried out on Illumina HiSeq2500 Analyzers (Illumina, SanDiego, USA. Following the manufacturer's standard sequencing protocols) for 90 cycles per read to generate paired‐end reads. Image analysis, error estimation, and base calling were performed using Illumina Pipeline software (version 1.3.4) to generate raw data.

As for quality control, the data were processed as follows: a. Discard paired reads if either one read contains adapter contamination (>10 nucleotides aligned to the adapter, allowing ≤10% mismatches); b. Discard paired reads if more than 10% of bases are uncertain in either read; c. Discard paired reads if the proportion of low‐quality bases (Phred quality <5) is over 50% in either read. All downstream bioinformatics analyses were based on high‐quality clean data.

The sequencing reads were aligned to the reference human genome (UCSC hg19) using BWA (Burrows‐Wheeler Aligner) Multi‐Vision software package.^9^ After alignment, the output files were used to perform sequencing coverage and depth analysis of the target region, single‐nucleotide variants (SNVs), and INDEL calling. We used SOAPsnp software ^10^ and Samtools ^11^ to detect SNVs and indels. All SNVs and indels were filtered and estimated via multiple databases, including NCBI dbSNP, HapMap, 1000 human genome dataset, and database of 100 Chinese healthy adults.^12^ Pathogenic variants were assessed under the protocol issued by the American College of Medical Genetics and Genomics (ACMG).^13^ The Human Gene Mutation Database (HGMD) was used to screen mutations reported in published studies.

#### 2.4.2 Verification of the variant

All mutations and potential pathogenic variants were validated by Sanger sequencing on an ABI3730 sequencer. The reference sequence of the WT1 gene is NM_024424.5.

### 2.5 In silico analysis

The mutation site was analyzed using Mutation Taster (http://www.mutationtaster.org/).^14^, ^15^, ^16^

### 2.6 Functional analysis of the novel variant

To detect the function of the novel *WT1* variant, we cloned the wild type and mutated coding DNA sequence (CDS) (NP_077742.3) of the *WT1* gene to pcDNA3.1 and compared their function by a cell growth inhibition assay.

#### 2.6.1 Plasmid construction

Total RNA was extracted from the tumor tissue using TRIzol® Reagent (Thermo Fisher Scientific). We used a QuantiTect reverse transcription kit (Qiagen) to synthesize cDNA. The full‐length CDS of the *WT1* gene was obtained by PCR with cDNA. The PCR primers were as follows: F‐5’‐GCGGATCCCTGGACTTCCTCTTGCTG‐3’, R‐5’‐GCGAATTCTCAAAGCGCCAGCTGGAG‐3’. To facilitate cloning, the PCR product and the vector (pcDNA3.1) were digested by BamHI and EcoRI. The PCR product was then inserted into pcDNA3.1. The pcDNA3.1‐*WT1* (C1420 T) was verified by Sanger sequencing. We analyzed the nucleotide sequences according to the NCBI Reference Sequence: NM_024424.5. The pcDNA3.1‐*WT1* (wild type) was generated by PCR‐based site‐directed mutagenesis. The primers used were as follows: F‐5’‐GACCACCTGAAGACCCACACCAGGACTCATA‐3’, R‐5’‐TATGAGTCCTGGTGTGGGTCTTCAGGTGGTC‐3’.

#### 2.6.2 Cell culture

G401 cells were used in this study. The cell line was purchased from the Cell Bank of the Chinese Academy of Sciences (Shanghai, China). The cells were grown in DMEM supplemented with 10% FBS and 1/1000 gentamycin.

#### 2.6.3 Cell transfection

Cell transfection was performed according to the protocol of Lipofectamine 2000 (Invitrogen; Thermo Fisher Scientific, Inc.) when cells reached 60% confluence. The cells were transfected with pcDNA3.1‐*WT1* (wild type), pcDNA3.1‐*WT1* (C1420 T), and the empty vector pcDNA3.1. The ratio of Lipofectamine 2000 to plasmid was 3:1. The transfected cells were digested with trypsin and seeded in 6‐well plates for western blotting, and 96‐well plate for cell growth inhibition assay.

#### 2.6.4 Western blot

After the transfected cells grew for 48 h in the 6‐well plate, we washed the cells with 1X PBS three times and lysed them with 100ul 1X RIPA per well. Thereafter, the protein concentration was quantified and 20 μg of proteins from each sample were segregated using 10% SDS‐PAGE gel. The samples were then transferred to PVDF membranes and incubated with primary antibodies (WT1: sc‐7385, Santa Cruz, 1:1000 dilution; GAPDH: sc‐47724, Santa Cruz, 1:1000 dilution) and secondary antibodies (m‐IgGκ BP‐HRP: sc‐516102, Santa Cruz, 1:2000 dilution). Finally, blots were detected with Chemiluminescent HRP Substrate reagent (Merck Millipore, Darmstadt, Germany), and the signal was captured by Bio‐Rad ChemiDoc XRS+ (BioRad Laboratories, Inc., Hercules, CA, USA).

#### 2.6.5 Cell growth inhibition assay

The cell growth inhibition assay was performed using the MTS method. We seeded 3000 transfected G401 cells from each group in 96‐well plates per well and cultured them for 7 days. Cell viability was tested using the MTS Cell Proliferation Assay kit (Promega) according to the standard protocol.

### 2.7 Statistical Analysis

Statistical analysis was performed using GraphPad Prism 8.0 (GraphPad, Inc.). The data are expressed as the mean ±standard deviation. Unpaired Student's t‐test was performed to evaluate differences between groups. *p* < 0.05 was considered to indicate a statistically significant difference. The experimental values are the means of triplicated independent repeats.

## 3 RESULTS

### 3.1 Clinical findings and post‐treatment follow‐up

#### 3.1.1 Physical examination

Physical examination revealed a palpable right abdominal mass with smooth margins. She also had elevated blood pressure (135/90 mmHg) and clitoral hypertrophy.

#### 3.1.2 Imageological examinations

**FIGURE 3:**
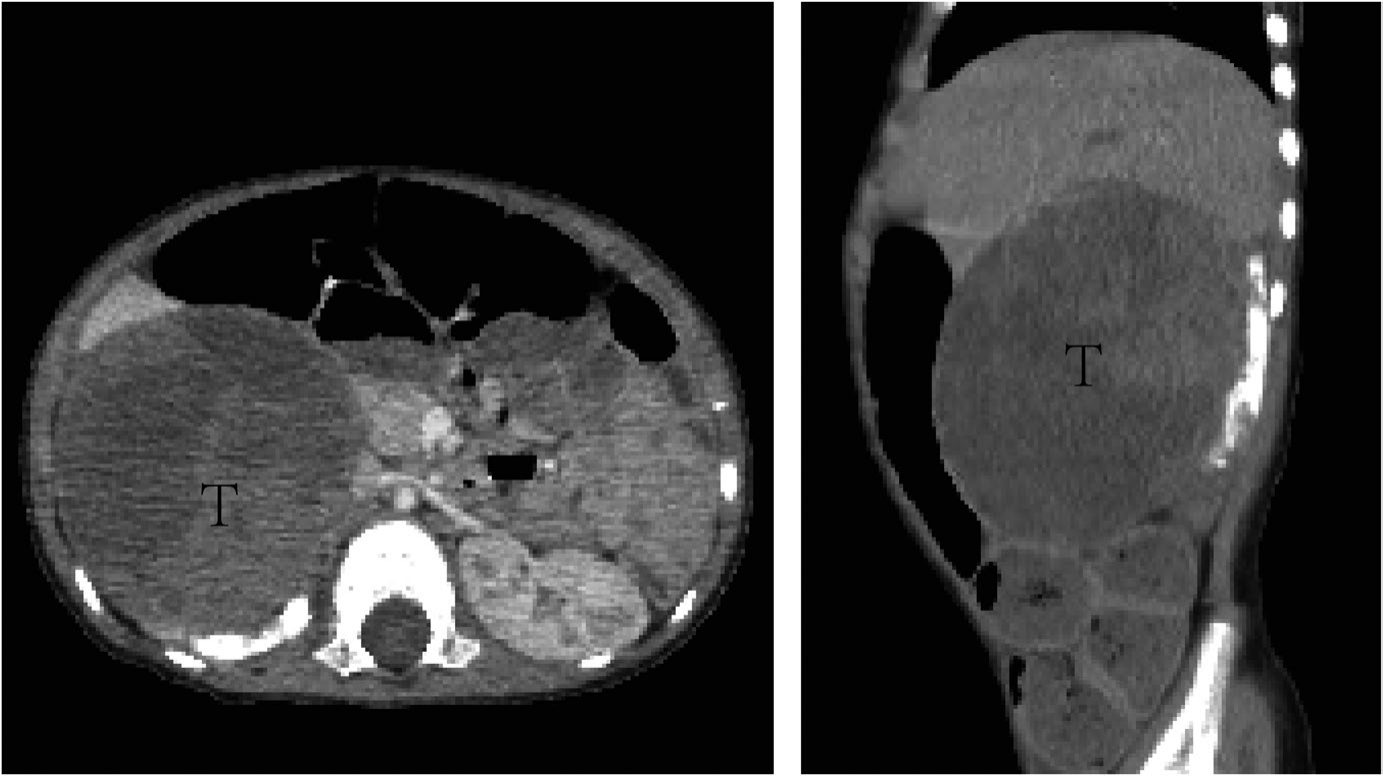
Contrast‐enhanced CT showing the 7.1 cm ×8.0 cm ×7.6 cm sized heterogeneous enhancing mass in the upper region of the right kidney. Left: transverse view of the tumor, Right: sagittal view of the tumor

Abdominal contrast‐enhanced CT revealed an approximately 7.1 × 8.0 × 7.6 cm heterogeneous enhancing mass in the upper region of the right kidney (Figure 3). Ultrasound imaging revealed that the vagina, uterus, and right ovary were almost normal. However, the left ovary was not clearly delineated.

#### 3.1.3 Laboratory tests and laparoscopic examination

**FIGURE 4:**
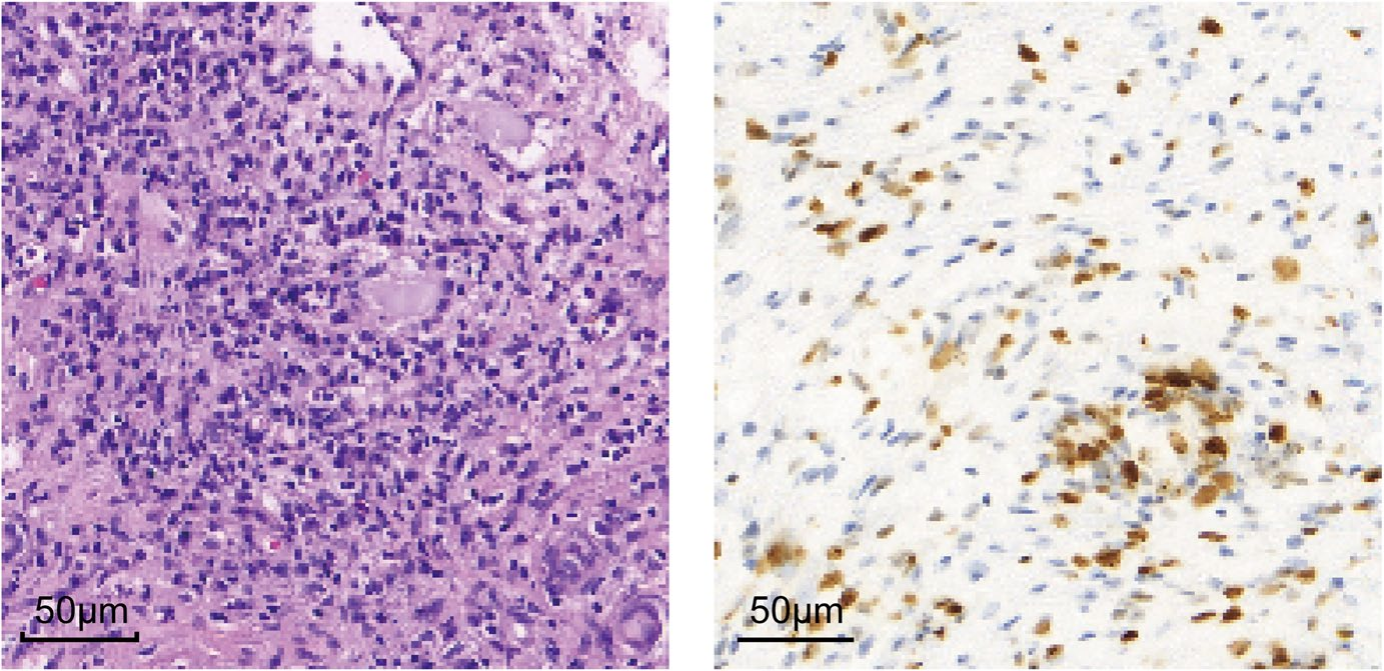
HE staining (left) and immunohistochemical staining (WT1) (right) after tumor resection

The patient had proteinuria without microscopic hematuria. She underwent a renal biopsy and histopathological examination indicated a diagnosis of Wilms tumor, and then, a right nephrectomy was performed. The pathologic report revealed a nephroblastoma (mesenchymal type) with necrosis based on the National Wilms Tumor Study Group 5 (NWTS‐5) classification criteria (Figure 4). The normal renal tissue structure was clear and demonstrated no evidence of glomerulosclerosis.

After completing chemotherapy and arterial interventional therapy, we evaluated the clitoral hypertrophy. The patient's vaginal and urethral orifices were grossly normal. However, her karyotype was 46, XY. Luteinizing hormone (LH) (1.8 IU/L; normal range 1.0–4.0 IU/L) was at a normal basal level, but follicle‐stimulating hormone (FSH) (128 IU/L; normal range 1.0–5.0 IU/L) was much higher than the normal basal level. Testosterone (< 0.01 ng/mL; normal range 0.07–0.52 ng/mL) was very low, and no elevation was observed after hCG stimulation.

To determine whether the gonads were normal, laparoscopy was performed when the patient was 29 months old. A small uterus and fallopian tube were observed during the laparoscopic examination. In addition, both gonads were located at the lateral side of the fallopian tube, so they were partly resected for biopsies. The pathology showed that both gonads were dysgenetic testicular tissue.

#### 3.1.4 Final Diagnosis and post‐treatment follow‐up

**FIGURE 5:**
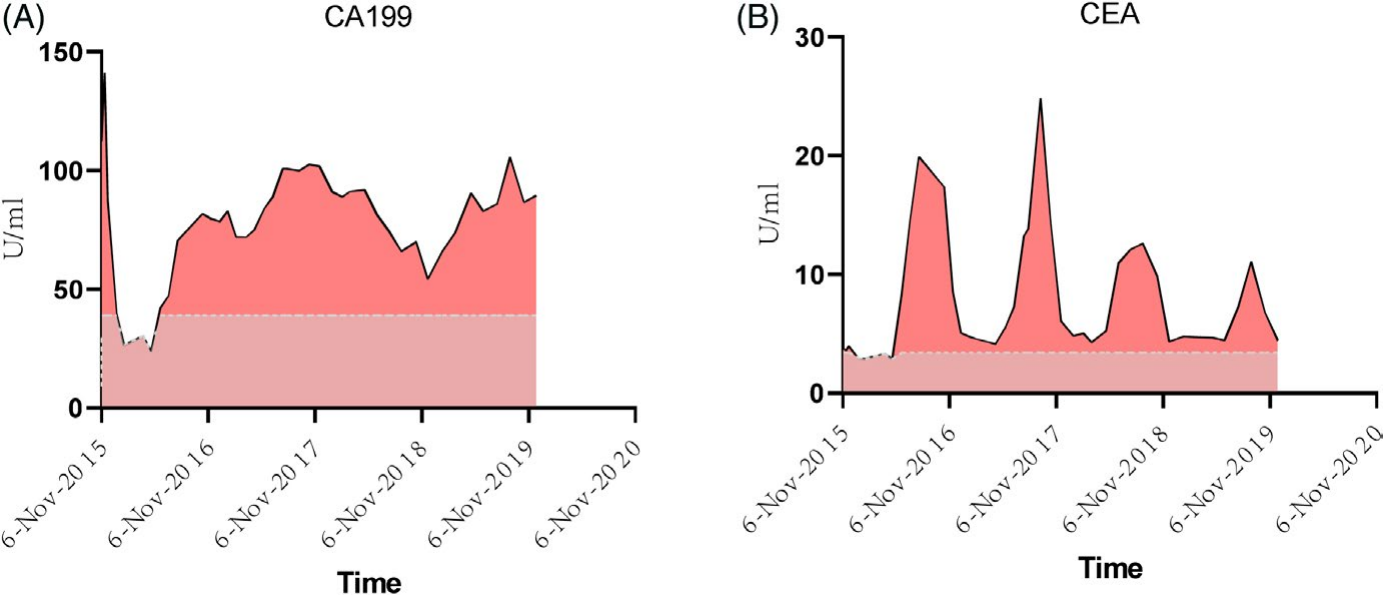
CA199. A, and CEA B, levels in peripheral blood in the follow‐up period. The full curve represents the measured value, and the dotted line represents the reference range. The last chemotherapy was on July 23, 2016

All clinical manifestations and molecular analyses demonstrated that the patient had Denys‐Drash syndrome. After 46 months of follow‐up, the patient was in good condition and showed no signs of tumor recurrence. However, the severity of the nephropathy increased and decreased. Albumin levels (1,030–3,226 mg/L; normal children <100 mg/L) were much higher than normal levels in the first two months, but her microalbuminuria was controlled well thereafter. Blood pressure was controlled using enalapril maleate and valsartan. In addition, CEA (2.89–24.85 U/ml; normal children <3.4 U/ml) and CA199 (3.31–141 U/ml; normal children <39 U/ml) were always higher than normal in the follow‐up period (Figure 5).

### 3.2 Identification of the novel mutation

Whole exon sequencing revealed that the *WT1* mutation was positive. The patient was found to have a heterozygous missense mutation, converting 474 His to 474 Tyr (c.1420C>T, Figure 1) in exon 9 of WT1, encoding a portion of the WT1 zinc‐finger 3 domain.

### 3.3 In silico analysis

**FIGURE 6:**
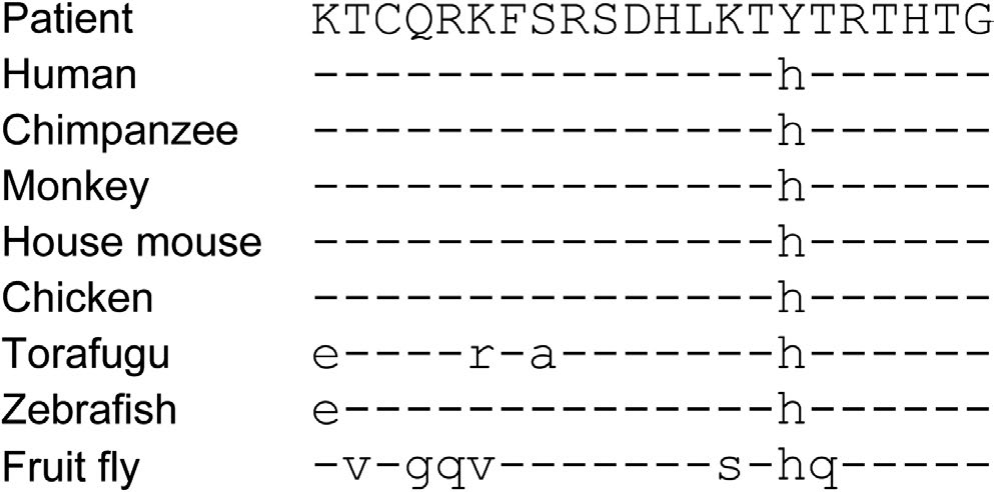
Multiple amino acid sequence alignment of the peptide encoded by WT1 of the patient with Human (Homo sapiens) (NCBI Reference Sequence: NM_024424.5), Chimpanzee (Pan troglodytes) (NCBI Reference Sequence: XM_016920639.2), Rhesus monkey (Macaca mulatta) (NCBI Reference Sequence: XM_028833535.1), House mouse (Mus musculus) (NCBI Reference Sequence: NM_144783.2), Chicken (Gallus gallus) (NCBI Reference Sequence: XM_015286134.2), Torafugu (Takifugu rubripes) (NCBI Reference Sequence: XM_011619095.2), Zebrafish (Danio rerio) (NCBI Reference Sequence: NM_131046.2), and Fruit fly (Drosophila melanogaster) (NCBI Reference Sequence: NM_001274762.1). Symbol (‐) represents the amino acids identical to those of the patient

The mutant variant of *WT1* (c.1420C>T, p. His474 Tyr) was predicted to “disease‐causing” by Mutation Taster. The peptide alignment revealed that the histidine in WT1 was a conserved residue (Figure 6).

### 3.4 Functional assays of the novel variant

**FIGURE 7:**
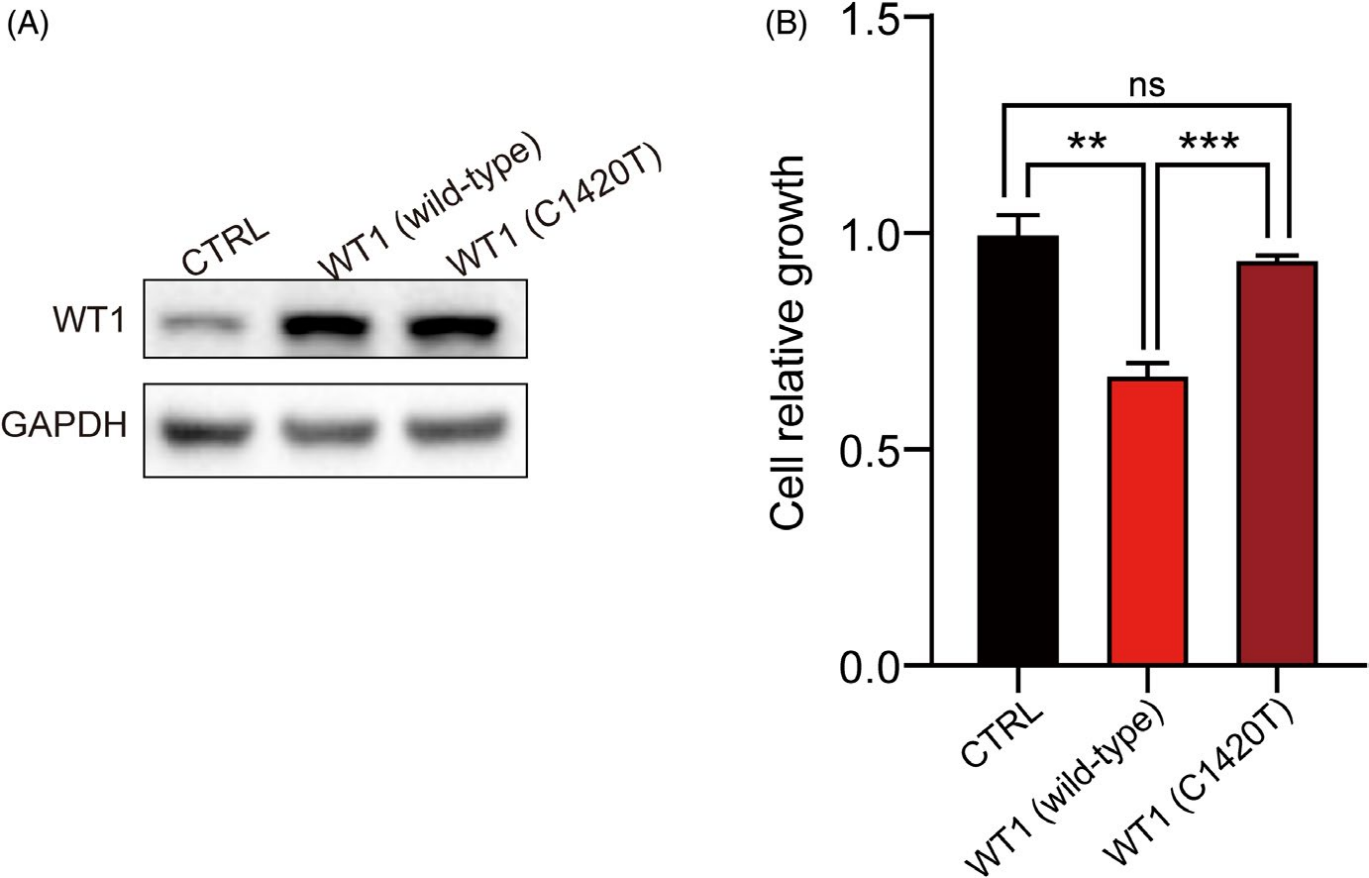
The function of WT1 (wild type and mutant variant) in G401 cells. A, The expression level of pcDNA3.1‐*WT1* (wild type) and pcDNA3.1‐*WT1* (C1420 T) was examined in a western blot analysis. B, Cell relative growth analysis. 3000 cells from each group were seeded in 96‐well plate per well and cultured for 7 days. Thereafter, we detected the cell relative growth rate by the MTS assay. All data are presented as the mean ±standard deviation from three independent experiments. Non‐paired t‐test was performed between the groups (**p < 0.05, ***p < 0.005, ns: non‐significance)

In the cell growth inhibition assay, we found that G401 cells with pcDNA3.1‐*WT1* (C1420 T) grew faster than those with pcDNA3.1‐*WT1* (wild type) but slower than the control group. Significant differences were observed between the groups (Figure 7). The result indicates that the mutant variant of *WT1* (c.1420C>T, p. His474 Tyr) cannot function as well as the wild‐type *WT1* as a tumor suppressor gene.

## 4 DISCUSSION

We present a case of a patient with Wilms tumor, nephropathy, hermaphroditism, and a novel mutation in the *WT1* gene. All the pathological features that this patient had met the typical characteristics were described in the cases of Denys‐Drash syndrome.^17^, ^18^

The human *WT1* gene is approximately 50 kb in length, spanning 10 exons.^19^, ^20^ The protein has four C2H2 zinc fingers (ZFs) for nucleic acid binding in the C‐terminal,^21^, ^22^ while its NH2 terminus contains both transcriptional repression and activation domains. It is a transcription factor with diverse functions in cells, ranging from organogenesis to tumor suppression.^23^ For this patient, we found a missense mutation in exon 9 (c.1420C>T, p. His474 Tyr) of the *WT1* gene that encodes ZF3. To the best of our knowledge, this is the first time this mutation is being reported in the literature.

In DDS, mutations in the ZF region can abolish the DNA‐binding capacity and lead to sex ambiguity because of dysgenetic testis, diffuse mesangial sclerosis with chronic renal disease, and a high incidence of Wilms tumor.^24^ In this study, we found that the histidine in WT1 was a conserved residue and that the mutant variant could not function well as a tumor suppression gene. In addition, the Mutation Taster tool also suggests that it is a “disease‐causing” mutant variant.

Mutant WT1 disrupts the development and differentiation of podocytes. The c.1420C>T mutation in exon 9 induces an amino acid change from histidine to tyrosine in ZF3. Histidine is an important component of the zinc‐finger structure. The binding affinity of the mutant peptides to the DNA target may become weaker. Studies have shown that WT1 activates the transcription of the podocalyxin gene.^25^, ^26^ The WT1 variant with a mutation in ZF9 may disrupt podocyte development and differentiation, leading to renal insufficiency. In mice with *WT1* knockout in podocytes, the podocyte foot processes disappeared and, consequently, glomerulosclerosis and proteinuria developed in mice. The results confirmed the essential role of *WT1* in podocyte maturation and maintenance.^27^, ^28^, ^29^ In addition, a study of histological specimens from children with DDS found that *WT1* expression in podocytes was abnormal in 80% of patients.^30^ For this patient, the mutation may play a crucial role in the process of nephrotic syndrome. Fortunately, the nephrotic syndrome was well controlled at the end of the follow‐up period. We will continue to monitor the development of nephropathy in the child closely.

In addition, WT1 also plays a key role in the regulation of SRY expression and sex determination. WT1 and steroidogenic factor 1 (SF‐1) are expressed in the urogenital ridge, which develops into the kidneys and gonads.^31^ WT1 associates with SF‐1 and achieves a synergistic effect. The upregulation of the SRY gene and the promotion of Müllerian inhibiting substance (MIS) both rely on the synergism of WT1 and SF‐1 expressed in the bipotential gonad.^32^, ^33^, ^34^ The mutant WT1 may disrupt the synergy between WT1 and SF‐1, which leads to gonadal abnormalities in 46, XY individuals.^35^, ^36^ This may explain the correlation between gonadal abnormalities and mutations in the *WT1* gene in this patient.

## 5 CONCLUSION

We report a novel mutation in *WT1* associated with DDS in a child. The uncommon missense mutation (c.1420C>T, p. His474 Tyr) in the *WT1* gene may be a crucial marker in DDS. WT1 sequencing should be considered early in children with sexual dysplasia, especially in patients with a 46, XY karyotype.

## CONFLICT OF INTERESTS

There is no conflict of interests.

## Data Availability

All data used for the analysis in this study are available in China National GeneBank DataBase (URL: https://db.cngb.org/cnsa/, Accession Number: CNP0001670).
